# Loss of the vitamin D receptor triggers senescence in chronic myeloid leukemia via DDIT4-mediated DNA damage

**DOI:** 10.1093/jmcb/mjad066

**Published:** 2023-10-25

**Authors:** Yan Xu, Wentao Qi, Chengzu Zheng, Yuan Li, Zhiyuan Lu, Jianmin Guan, Chunhua Lu, Baobing Zhao

**Affiliations:** Key Lab of Chemical Biology (MOE), School of Pharmaceutical Sciences, Cheeloo College of Medicine, Shandong University, Jinan 250012, China; NMPA Key Laboratory for Technology Research and Evaluation of Drug Products, School of Pharmaceutical Sciences, Cheeloo College of Medicine, Shandong University, Jinan 250012, China; Department of Pharmacology, School of Pharmaceutical Sciences, Cheeloo College of Medicine, Shandong University, Jinan 250012, China; Key Lab of Chemical Biology (MOE), School of Pharmaceutical Sciences, Cheeloo College of Medicine, Shandong University, Jinan 250012, China; NMPA Key Laboratory for Technology Research and Evaluation of Drug Products, School of Pharmaceutical Sciences, Cheeloo College of Medicine, Shandong University, Jinan 250012, China; Department of Pharmacology, School of Pharmaceutical Sciences, Cheeloo College of Medicine, Shandong University, Jinan 250012, China; Key Lab of Chemical Biology (MOE), School of Pharmaceutical Sciences, Cheeloo College of Medicine, Shandong University, Jinan 250012, China; NMPA Key Laboratory for Technology Research and Evaluation of Drug Products, School of Pharmaceutical Sciences, Cheeloo College of Medicine, Shandong University, Jinan 250012, China; Department of Pharmacology, School of Pharmaceutical Sciences, Cheeloo College of Medicine, Shandong University, Jinan 250012, China; Key Lab of Chemical Biology (MOE), School of Pharmaceutical Sciences, Cheeloo College of Medicine, Shandong University, Jinan 250012, China; NMPA Key Laboratory for Technology Research and Evaluation of Drug Products, School of Pharmaceutical Sciences, Cheeloo College of Medicine, Shandong University, Jinan 250012, China; Department of Pharmacology, School of Pharmaceutical Sciences, Cheeloo College of Medicine, Shandong University, Jinan 250012, China; School of Pharmaceutical Sciences & Institute of Materia Medica, Shandong First Medical University & Shandong Academy of Medical Sciences, Jinan 250012, China; Department of Hematology, Heze Municipal Hospital, Heze 274031, China; Key Lab of Chemical Biology (MOE), School of Pharmaceutical Sciences, Cheeloo College of Medicine, Shandong University, Jinan 250012, China; NMPA Key Laboratory for Technology Research and Evaluation of Drug Products, School of Pharmaceutical Sciences, Cheeloo College of Medicine, Shandong University, Jinan 250012, China; Key Lab of Chemical Biology (MOE), School of Pharmaceutical Sciences, Cheeloo College of Medicine, Shandong University, Jinan 250012, China; NMPA Key Laboratory for Technology Research and Evaluation of Drug Products, School of Pharmaceutical Sciences, Cheeloo College of Medicine, Shandong University, Jinan 250012, China; Department of Pharmacology, School of Pharmaceutical Sciences, Cheeloo College of Medicine, Shandong University, Jinan 250012, China

**Keywords:** vitamin D receptor, senescence, chronic myeloid leukemia, DDIT4

## Abstract

Chronic myeloid leukemia (CML) is a hematopoietic malignancy driven by the fusion gene *BCR*::*ABL1*. Drug resistance to tyrosine kinase inhibitors (TKIs), due to BCR::ABL1 mutations and residual leukemia stem cells (LSCs), remains a major challenge in CML treatment. Here, we revealed the requirement of the vitamin D receptor (VDR) in the progression of CML. VDR was upregulated by BCR::ABL1 and highly expressed in CML cells. Interestingly, VDR knockdown inhibited the proliferation of CML cells driven by both BCR::ABL1 and TKI-resistant BCR::ABL1 mutations. Mechanistically, VDR transcriptionally regulated *DDIT4* expression; reduced DDIT4 levels upon VDR knockdown triggered DNA damage and senescence via p53 signaling activation in CML cells. Furthermore, VDR deficiency not only suppressed tumor burden and progression in primary CML mice but also reduced the self-renewal capacity of CML-LSCs. Together, our study demonstrated that targeting VDR is a promising strategy to overcome TKI resistance and eradicate LSCs in CML.

## Introduction

Chronic myeloid leukemia (CML) is a hematopoietic malignancy in which hematopoietic stem cells (HSCs) are transformed by the *BCR::ABL1* fusion gene generated from a reciprocal translocation [t(9;22)(q34;q11.2)] (Cortes et al., 2021). This fusion gene encodes the BCR::ABL1 protein with constitutively activated tyrosine kinase, resulting in the malignant expansion of myeloid cells in the bone marrow (BM) and peripheral blood (Druker, 2008). The successful application of tyrosine kinase inhibitors (TKIs) targeting the adenosine triphosphate binding site of BCR::ABL1, such as imatinib (IM), has greatly improved the event-free survival of CML patients (Ma et al., 2014; Kalmanti et al., 2015). However, acquired resistance due to emerging BCR::ABL1 kinase domain mutations enforced the development of second- and third-generation TKIs (Braun et al., 2020) and will continue to drive the development of novel TKIs (O’Hare et al., 2012; Saikia, 2018). Therefore, strategies aiming to inhibit BCR::ABL1 signaling despite its mutations are of increased importance for the functional cure of CML (Loscocco et al., 2019). In this study, we found that the vitamin D receptor (VDR) is upregulated by BCR::ABL1 in CML, independent of its clinical mutations.

VDR is a transcriptional regulator belonging to the superfamily of nuclear steroid/thyroid hormone receptors. It mediates most of the genomic actions of the active metabolite of vitamin D, 1,25-dihydroxy-vitamin D [1,25(OH)2D]. Upon ligand binding, VDR forms a heterodimer with the retinoid X receptor to bind to vitamin D-responsive elements of target genes, playing key roles in numerous biological processes (Deeb et al., 2007; Medrano et al., 2018). Due to its altered expression and function, the role of VDR in cancers has also been extensively studied (Campbell and Trump, 2017). Vitamin D-mediated VDR signaling activation exhibits antiproliferative effects against multiple cancers (Larriba et al., 2011; Li et al., 2012; Zhang et al., 2020; Ling et al., 2022). However, there is also increasing evidence to support an oncogenic role for VDR in tumor progression. VDR knockdown inhibited the growth of glioblastoma and T-ALL cells (Shirvani-Farsani and Behmanesh, 2019), and lung metastatic cancer growth was extremely reduced in VDR-null mice (Nakagawa et al., 2004). Loss of VDR eliminated breast and prostate cancers through downregulation of Wnt/β-catenin signaling (Zheng et al., 2017). Furthermore, VDR functions as a master regulator of MYCN, which is highly expressed in over 70% of malignancies (Salehi-Tabar et al., 2012; Liu et al., 2020). These findings indicated that the distinct roles of VDR in cancers depend on the tumor cell type or whether it is independent of its ligand.

VDR expression and activity have been documented to be associated with several types of leukemia, such as acute lymphoblastic leukemia (ALL) and acute myelocytic leukemia (AML) (Pezeshki et al., 2018). Targeting VDR provides antileukemic activity by acting on cell differentiation and decreasing the stemness of AML cells (Paubelle et al., 2020). Accordingly, vitamin D and its analogs have been considered potent drugs for AML therapy via VDR activation (Paubelle et al., 2020; Sabatier et al., 2021). However, the role of VDR in CML is still incompletely described. In this study, we present an unexpected role of VDR in the progression of CML. Targeting VDR provides an effective therapy for CML, representing a novel therapeutic target for overcoming resistance to TKIs.

## Results

### VDR is upregulated by the fusion gene *BCR::ABL1* in CML

**Figure 1:**
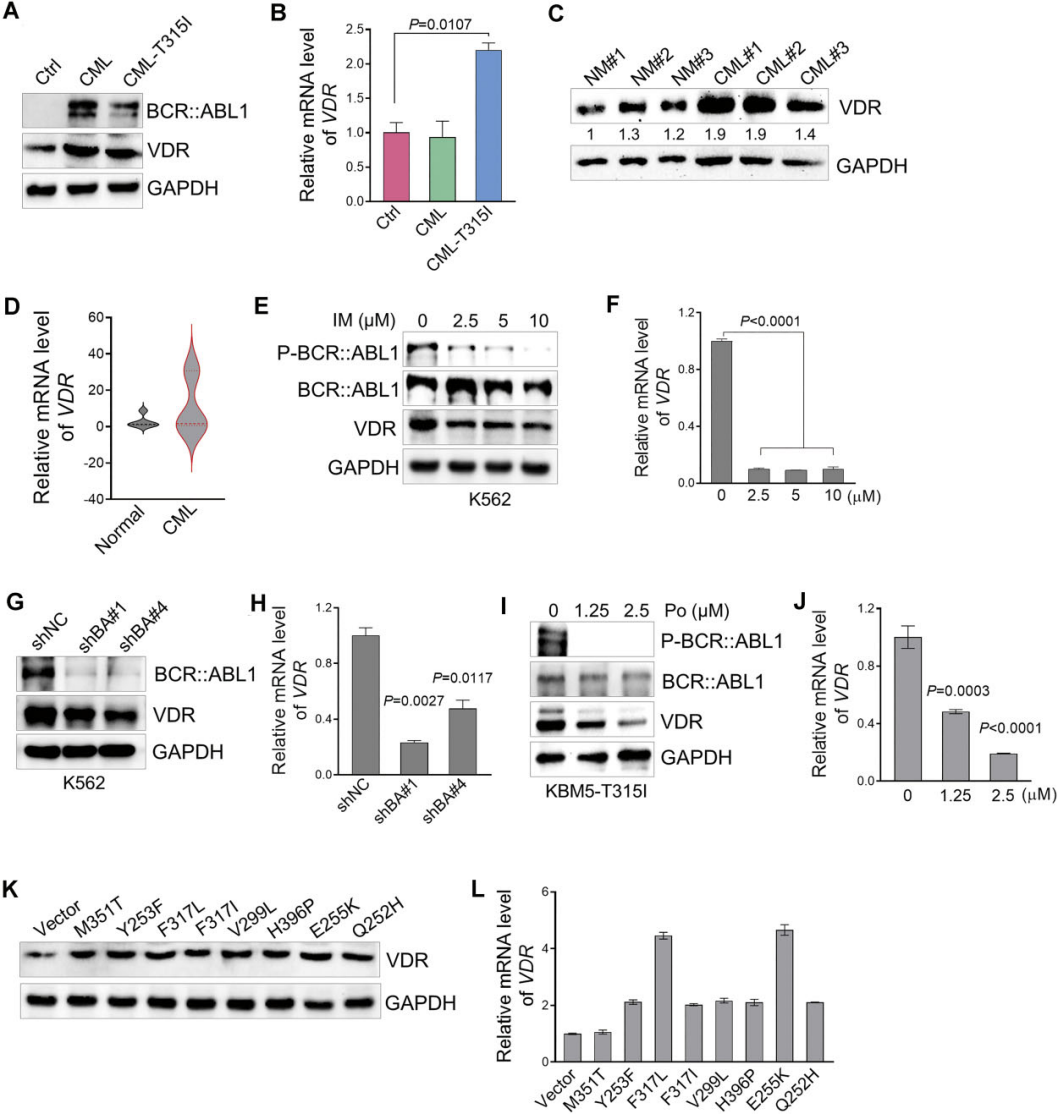
VDR is highly expressed in CML cells, and BCR::ABL1 regulates VDR expression. (**A** and **B**) Immunoblotting analysis of VDR and BCR::ABL1 protein levels (**A**) and relative *VDR* mRNA levels (**B**) in BM cells from the indicated CML mice and corresponding controls (Ctrl). GAPDH was used as a loading control. (**C** and **D**) Immunoblotting analysis of VDR protein levels (**C**) and relative *VDR* mRNA levels (**D**) in BM cells from CML patients and healthy donors (normal, NM). GAPDH was used as a loading control. (**E** and **F**) Immunoblotting analysis of the indicated protein levels (**E**) and relative *VDR* mRNA levels (**F**) in K562 cells treated with IM for 10 h. GAPDH was used as a loading control. (**G** and **H**) Immunoblotting analysis of VDR and BCR::ABL1 proteins (**G**) and relative *VDR* mRNA levels (**H**) in K562 cells transduced with retroviruses encoding the indicated shRNA. shNC represents a nontargeting shRNA; shBA represents a shRNA targeting BCR::ABL1. GAPDH was used as a loading control. (**I** and **J**) Immunoblotting analysis of the indicated proteins (**I**) and relative *VDR* mRNA levels (**J**) in KBM5-T315I cells treated with Po for 10 h. GAPDH was used as a loading control. (**K** and **L**) Immunoblotting analysis of VDR protein levels (**K**) and relative VDR mRNA levels (**L**) in Ba/F3 cells transduced with retroviruses encoding the indicated BCR::ABL1 mutations or empty vector. GAPDH was used as a loading control. All *P* values were determined by an unpaired two-tailed Student's *t*-test except where indicated. Data are presented as mean ± SD from three independent experiments. See also Supplementary Figure S1.

To explore the potential role of VDR in CML, we used a well-established BM transplantation model with ectopic expression of BCR::ABL1 (referred to as BCR::ABL1-driven CML mice), which mimics the pathogenesis in human CML patients. Immunoblotting analysis demonstrated that VDR was upregulated in BM cells from BCR::ABL1-driven CML mice compared with that of the control group, whereas the mRNA level of *VDR* showed no detectable changes in either group of mice (Figure 1A and B). In line with these findings, similar upregulation of VDR was observed in Ba/F3 cells with ectopic expression of BCR::ABL1 (referred to as Ba/F3^BCR^^::^^ABL1^ cells) (Supplementary Figure S1A and B). Importantly, the protein but not mRNA levels of VDR were also significantly upregulated in BM samples from CML patients compared with the corresponding healthy donors (Figure 1C and D). These data suggest that BCR::ABL1 regulates VDR expression in CML.

We then challenged K562 and KBM5, two CML cell lines harboring the fusion gene *BCR::ABL1*, with IM, which specifically inhibits the tyrosine kinase activity of BCR::ABL1. As expected, VDR expression was notably reduced by IM treatment (Figure 1E and F; Supplementary Figure S1C and D). Furthermore, silencing BCR::ABL1 with shRNAs also led to the downregulation of VDR in K562 cells (Figure 1G and H). Considering that the protein but not mRNA level of VDR was greatly upregulated in Ba/F3^BCR^^::^^ABL1^ cells, we challenged these cells with cycloheximide (CHX) to inhibit protein synthesis and found that BCR::ABL1 overexpression led to the retaining abundance of VDR compared to that of control cells (Supplementary Figure S1E and F). These findings demonstrated that BCR::ABL1 regulated *VDR* mRNA expression and stabilized VDR.

BCR::ABL1^T315I^ is a common point mutation resistant to all earlier-generation TKIs, remaining one major obstacle to vanquishing CML (Braun et al., 2020; Hughes and Shanmuganathan, 2022). Interestingly, we found that VDR was also highly expressed in BCR::ABL1^T315I^-driven CML mice and Ba/F3 cells (Figure 1A and B; Supplementary Figure S1A and B). Both the protein and mRNA levels of VDR were dramatically reduced in KBM5-T315I cells harboring the IM-resistant BCR::ABL1^T315I^ mutation by treatment with ponatinib (Po), which efficiently inhibited the activity of BCR::ABL1^T315I^ (Figure 1I and J). We also challenged Ba/F3^T315I^ cells with CHX but found that BCR::ABL1^T315I^ overexpression led to similar changes in VDR protein levels compared to those in control cells (Supplementary Figure S1G and H).

To further confirm that VDR expression is regulated by BCR::ABL1 independent of its mutations, we generated corresponding Ba/F3 cells stably expressing several documented drug-resistant BCR::ABL1 mutations and found that VDR exhibited high expression in these mutant BCR::ABL1 compared with control cells (Figure 1K and L). Notably, similar upregulation of VDR was also observed in Ba/F3 cells with ectopic expression of BCR::ABL1^E255V+T315I^, which confers resistance to Po (Supplementary Figure S1I and J). These data demonstrated that VDR expression is upregulated in CML independent of BCR::ABL1 mutations.

### VDR knockdown inhibits CML cell proliferation

**Figure 2:**
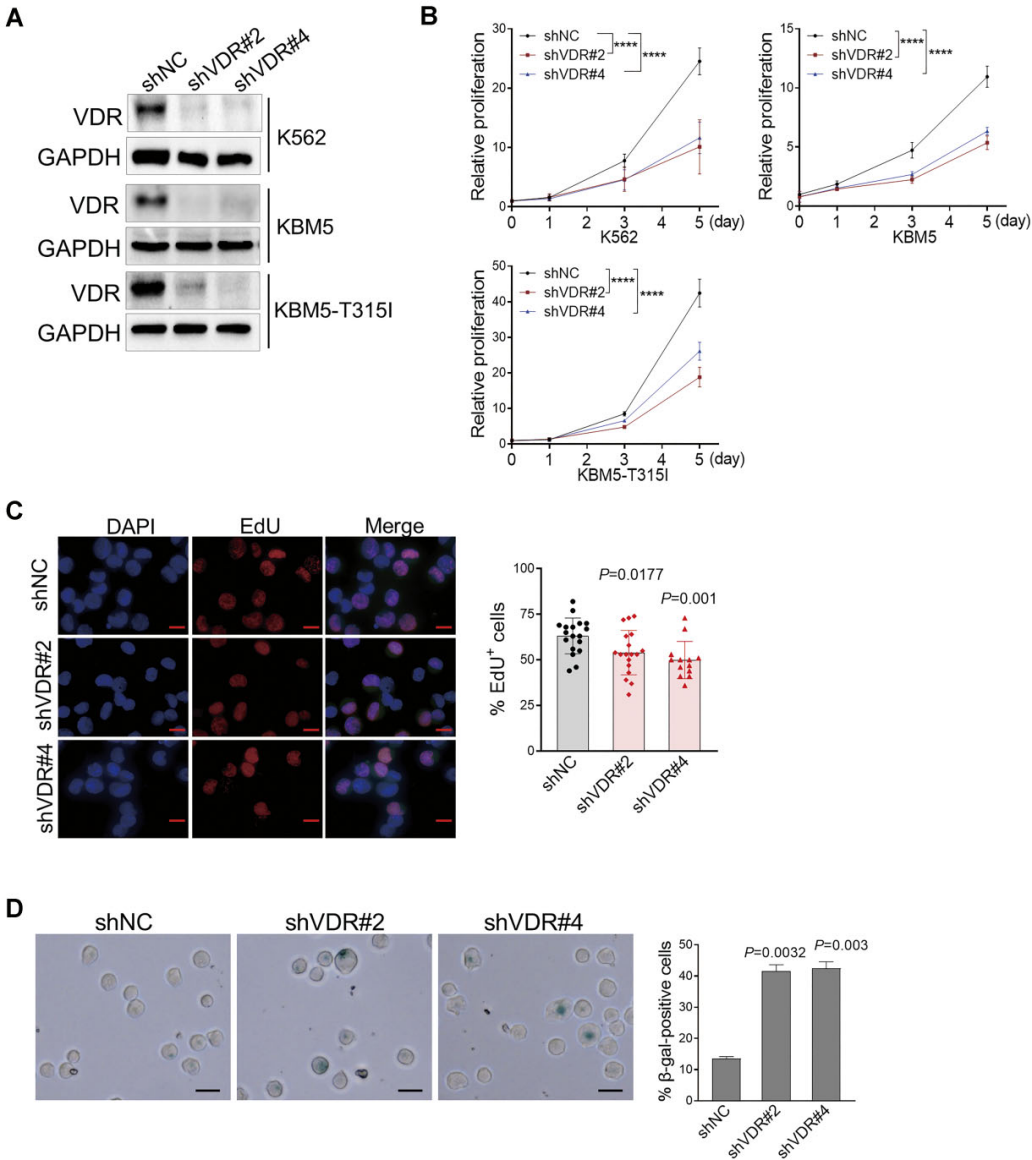
VDR knockdown inhibits CML cell proliferation. (**A**) Immunoblotting analysis of VDR protein levels in K562, KBM5, and KBM5-T315I cells transduced with retroviruses encoding the indicated shRNA. shNC represents a nontargeting shRNA. GAPDH was used as a loading control. (**B**) Statistical analysis of cell proliferation in cells as in **A**. Data were obtained from three independent experiments. The *P* value was determined by two-way ANOVA. ****P < 0.0001. (**C**) K562 cells transduced with retroviruses encoding the indicated shRNA were labeled with EdU for 2 h and stained with azide-conjugated Alexa567 (red) and DAPI (blue). shNC represents a nontargeting shRNA. Scale bar, 200 μm. Quantification of the percentage of EdU-positive cells is shown on the right. (**D**) Representative images of SA-β-gal staining in K562 cells transduced with retroviruses encoding the indicated shRNAs. shNC represents a nontargeting shRNA. Quantitation of the percentage of SA-β-gal-positive cells in the indicated group is shown on the right. Scale bar, 25 μm. All *P* values were determined by an unpaired two-tailed Student's *t*-test except where indicated. See also Supplementary Figure S2.

To examine whether high expression of VDR is involved in the progression of CML, we first performed proliferation assays in K562 cells transduced with retrovirus-encoding VDR shRNA. Indeed, we examined BCR::ABL1 expression in K562 cells with VDR silencing and found that VDR knockdown had no effect on BCR::ABL1 (Supplementary Figure S2A and B). Compared to the control group, VDR silencing led to substantial inhibition of K562 cell proliferation (Figure 2A and B). This was further determined by the repressed DNA replication indicated by the reduced EdU staining in K562 cells upon VDR knockdown (Figure 2C). Conversely, overexpression of VDR led to a mild increase in K562 cell growth (Supplementary Figure S2C and D).

To explore the underlying mechanism by which VDR regulates CML cell proliferation, we analyzed the cell cycle profile of K562 cells and found that VDR knockdown had minimal effects on the cell cycle (Supplementary Figure S2E). In addition, K562 cells with VDR silencing also exhibited comparable cell viability to that of the control group (data not shown). Since cellular senescence is characterized by permanent proliferation arrest, we performed a senescence-associated-β-galactosidase (SA-β-gal, a biomarker of senescence) staining assay and found that VDR knockdown triggered senescence, as indicated by the marked increase in SA-β-gal-positive K562 cells (Figure 2D).

Compound mutations in BCR::ABL1 contribute to resistance to TKIs in CML patients, which remains a clinical challenge for CML therapy (Cortes et al., 2021). Interestingly, similar inhibition of cell proliferation induced by VDR knockdown was also observed in KBM5 and IM-resistant KBM5-T315I cells (Figure 2A and B). In line with the upregulation of VDR in BCR::ABL1 mutation-driven Ba/F3 cells, VDR knockdown significantly inhibited the proliferation of all these transformed CML cells (Supplementary Figure S2F). Importantly, even Po-resistant mutation-driven proliferation was dramatically reduced by VDR knockdown in Ba/F3 cells (Supplementary Figure S2G). These data demonstrated that inhibiting VDR effectively suppressed the proliferation of CML cells independent of BCR::ABL1 mutations, avoiding the resistance of CML to TKIs.

To examine whether VDR depletion increases CML cell sensitivity to TKIs, we performed proliferation assays in KBM5 and KBM5-T315I cells transduced with retrovirus-encoding VDR shRNA, followed by treatment with IM and Po, respectively. Compared to the corresponding controls, both TKIs showed similar inhibition of CML cell proliferation upon VDR silencing. However, combined VDR knockdown and TKIs exhibited synergistic inhibition of CML cell proliferation (Supplementary Figure S2H).

### VDR knockdown triggers senescence in CML cells via DDIT4-mediated DDR signaling of γ-H2AX/p53/p21

**Figure 3:**
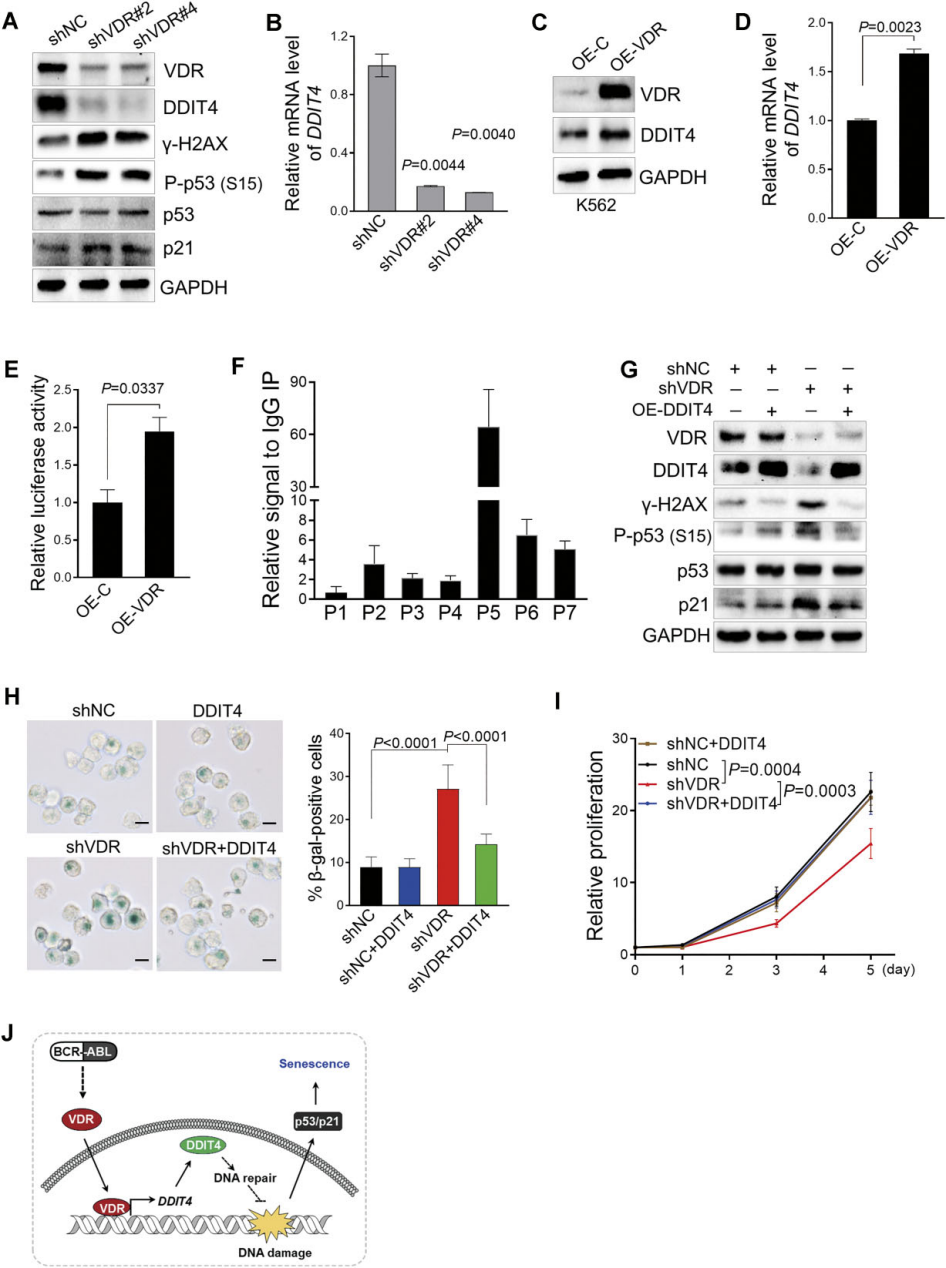
VDR knockdown triggers cell senescence in CML cells via DDIT4-mediated DDR signaling of γ-H2AX/p53/p21. (**A** and **B**) Immunoblotting analysis of the indicated protein levels (**A**) and relative *DDIT4* mRNA levels (**B**) in K562 cells transduced with retroviruses encoding shVDR or shNC. shNC represents a nontargeting shRNA. GAPDH was used as a loading control. (**C** and **D**) Immunoblotting analysis of VDR and DDIT4 protein levels (**C**) and relative *DDIT4* mRNA levels (**D**) in K562 cells transduced with retroviruses encoding VDR or emptyvector (OE-C). GAPDH was used as a loading control. (**D**) Quantification of *DDIT4* mRNA expression in cells as in **C**. (**E**) Luciferase reporter assays of the *DDIT4* promoter in HEK293T cells cotransfected with a luciferase reporter construct bearing the –2000 *DDIT4* promoter and a VDR plasmid or empty vector (OE-C). Data are presented as mean ± SD from three independent experiments. (**F**) Chromatin immunoprecipitation analyses of VDR binding to the *DDIT4* promoter in K562 cells. P1–P7 indicate fragments in the DDIT4 region amplified in ChIP‒qPCR assays (see also Supplementary Figure S3E). (**G**) Immunoblotting analysis of the indicated protein levels in K562 cells transduced with retroviruses encoding shVDR or shNC and DDIT4. shNC represents a nontargeting shRNA. GAPDH was used as a loading control. (**H**) Ectopic expression of DDIT4 rescued VDR knockdown-induced senescence in K562 cells. Representative images of SA-β-gal staining are shown on the left. Quantitation of the percentage of SA-β-gal-positive cells in the indicated group is shown on the right. Scale bar, 12.5 μm. (**I**) Statistical analysis of cell proliferation in cells as in **G**. Data were obtained from three independent experiments. The *P* value was determined by two-way ANOVA. * *P *< 0.05, *** *P *< 0.001. (**J**) A schematic model depicting the role of the VDR–DDIT4–p53 axis in CML cells. All *P* values were determined by an unpaired two-tailed Student's *t*-test except where indicated. See also Supplementary Figure S3.

To understand the molecular mechanism underlying the cellular senescence induced by VDR silencing, we performed bulk RNA sequencing of K562 cells transduced with retrovirus-encoding VDR shRNA. A total of 1502 differentially expressed genes (DEGs) were found (≥1.2-fold, *P *< 0.05), including 639 upregulated and 863 downregulated genes (Supplementary Figure S3A). Enrichment analysis showed that these DEGs were involved in DNA damage, cell cycle regulation, and p53 signaling (Supplementary Figure S3B and Table S1). Consistently, immunoblotting analysis demonstrated that VDR knockdown led to the elevated phosphorylation of H2AX, a well-known DNA damage marker, accompanied by increased p53 phosphorylation and p21 expression (Figure 3A). Furthermore, p53 inhibition significantly recovered the senescence and cell proliferation inhibition driven by VDR knockdown (Supplementary Figure S3C and D), suggesting that VDR knockdown triggered DNA damage response (DDR) signaling, which consequently resulted in senescence in K562 cells.

Among these DEGs, DNA damage-inducible transcript 4 (*DDIT4*), known to be induced by various cellular stresses (Ellisen et al., 2002), was downregulated upon VDR knockdown. This was further confirmed by its reduced protein and mRNA levels in K562 cells transduced with VDR shRNA compared to the control group (Figure 3A and B). In contrast, VDR overexpression resulted in a remarkable increase in the protein and mRNA levels of DDIT4 in K562 cells (Figure 3C and D). Considering that VDR is a member of the nuclear receptor superfamily and regulates the expression of numerous genes, we directly explored whether DDIT4 is transcriptionally regulated by VDR. A luciferase assay showed that ectopic expression of VDR markedly increased luciferase activity in HEK293T cells transduced with the *DDIT4* promoter and VDR (Figure 3E). We also performed a chromatin immunoprecipitation assay to validate the binding of VDR with the DDIT4 promoter. By sequentially using ChIP followed by quantitative PCR, we found that VDR was preferentially bound to the regions most distal to the start codon of DDIT4 (Figure 3F; Supplementary Figure S3E). These data demonstrated that *DDIT4* is a transcriptional target of VDR.

To assess the role of DDIT4 in CML cell proliferation mediated by VDR, we first examined DDIT4 expression in CML cells. Immunoblotting analysis demonstrated that DDIT4 was upregulated in BM cells from both BCR::ABL1-driven and BCR::ABL1^T315I^-driven CML mice compared to the corresponding control group (Supplementary Figure S3F). Similarly, high expression of DDIT4 was also observed in BM samples from CML patients compared to the corresponding healthy donors (Supplementary Figure S3G and H). In addition, the high expression of DDIT4 was dramatically abolished by IM treatment (Supplementary Figure S3I and J). We then performed rescue experiments in K562 cells and found that enforced expression of DDIT4 obviously restored the activated DDR signaling of γ-H2AX/p53/p21 induced by VDR knockdown (Figure 3G). As a downstream consequence, cellular senescence and subsequent cell proliferation inhibition were also successfully recovered by DDIT4 in VDR-silenced K562 cells (Figure 3H and I). Taken together, these data demonstrated that loss of VDR triggers senescence in CML cells via DDIT4-mediated DDR signaling of γ-H2AX/p53/p21 (Figure 3J).

### Loss of VDR ameliorates BCR::ABL1-driven CML phenotypes

To clarify the functional roles of VDR in the progression of BCR::ABL1-driven CML, we utilized a VDR knockout (KO) mouse model, in which VDR deficiency was confirmed by the undetectable expression of VDR in BM cells compared to the wild-type (WT) control (Supplementary Figure S4A and B). Indeed, KO mice exhibited comparable blood cell counts in peripheral blood and BM, excluding a minor decrease in platelets (Supplementary Figure S4C and D), as well as a similar frequency of hematopoietic stem and progenitor cells (HSPCs) in BM compared to that of WT controls (Supplementary Figure S4E). These data indicated that VDR does not contribute to steady-state hematopoiesis *in vivo*.

**Figure 4:**
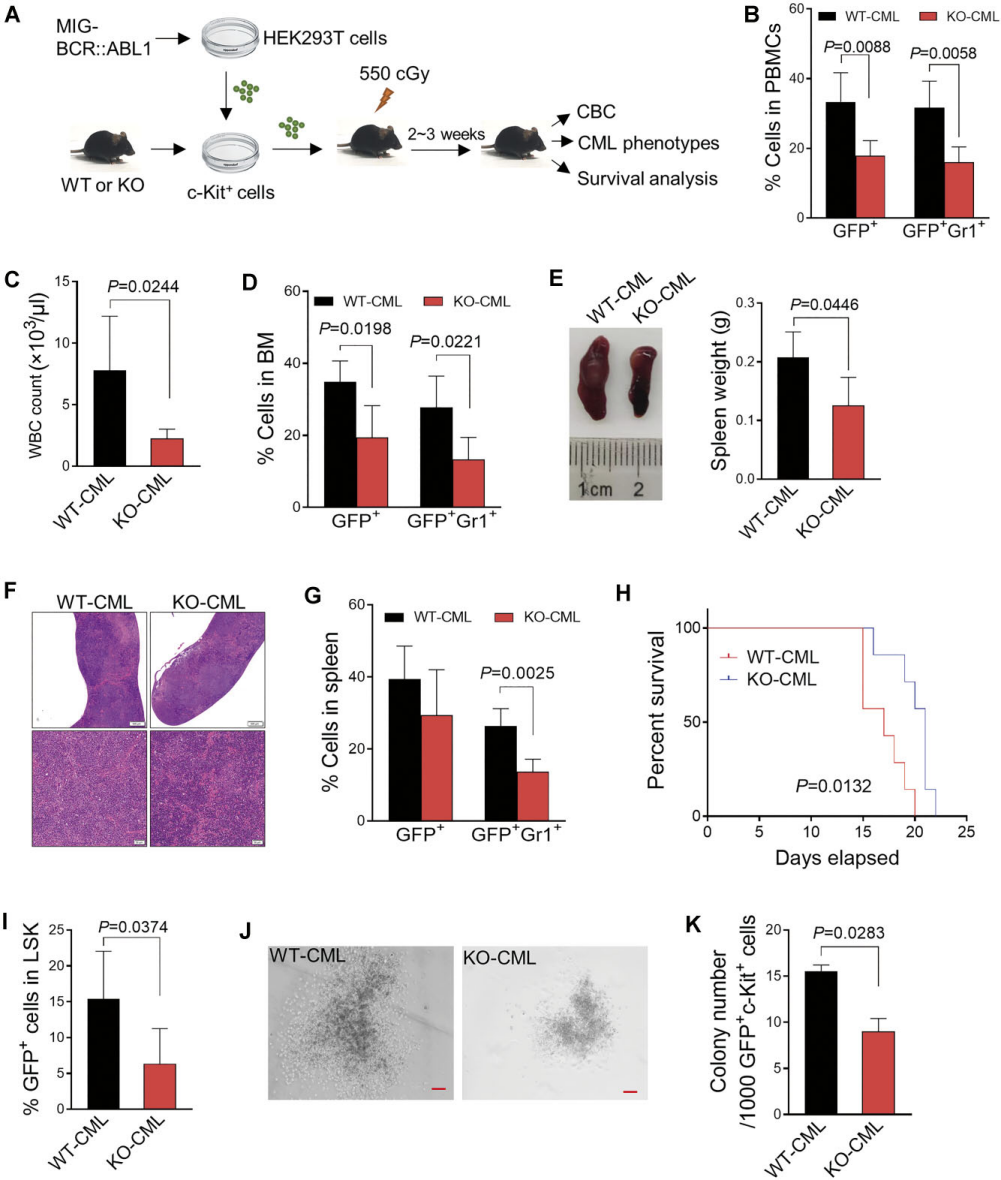
Loss of VDR ameliorates BCR::ABL1-driven CML phenotypes. (**A**) Schematic representation of the BCR::ABL1-driven CML mouse model with WT or VDR KO mouse BM cells. CBC represents complete blood count. (**B**) Quantification of the percentage of GFP^+^ cells and GFP^+^Gr1^+^ cells in peripheral blood mononuclear cells (PBMCs) from the mice described in **A** (*N* = 5). (**C**) White blood cells (WBCs) in peripheral blood were measured 14 days after transplantation from the indicated mice as in **A** (*N* = 5). (**D**) Quantification of the percentage of GFP^+^ cells and GFP^+^Gr1^+^ cells in the BM from the indicated CML mice measured by flow cytometry (*N* = 5). (**E**) Representative spleens of the indicated CML mice described in **A**. Statistical analysis of spleen weight is shown on the right (*N* = 4). (**F**) Representative hematoxylin and eosin (H&E) staining of spleens from the indicated CML mice 14 days after transplantation. Upper scale bar, 500 μm, lower scale bar, 50 μm. (**G**) Quantification of the percentage of GFP^+^ cells and GFP^+^Gr1^+^ cells in the spleen from the indicated mice measured by flow cytometry (*N* = 5). (**H**) Kaplan–Meier survival curves of the indicated CML mice as in **A** (*N* = 8). *P* values were determined by the log-rank (Mantel–Cox) test. (**I**) Quantification of the percentage of GFP^+^LSK (Lin^–^Sca1^+^c-Kit^+^) in the BM from the indicated mice measured by flow cytometry (*N* = 5). (**J**) Representative colonies of GFP^+^c-Kit^+^ BM cells from the indicated CML mice. Scale bar, 10 μm. (**K**) Quantification of the colony number in the indicated group as in **J**. All *P* values were determined by an unpaired two-tailed Student's *t*-test except where indicated. See also Supplementary Figure S4.

We then utilized a CML mouse model to assess the impact of VDR deletion on the disease, in which VDR-KO or WT BM was transduced with BCR::ABL1 and then transplanted into irradiated recipient mice (referred to as WT-CML and KO-CML mice) (Figure 4A). Homing assays demonstrated that VDR deficiency did not affect the engraftment of HSCs (Supplementary Figure S4F). The leukemia burden was evaluated by assessing the degree of organ lesions and CML cell infiltration. As previously reported (Peng and Li, 2016), the most common blast crisis in CML is myeloid (Supplementary Figure S4G). KO-CML mice showed a substantial reduction in leukemic cells (GFP^+^Gr1^+^) in the peripheral blood and BM after 14 days of CML model establishment, accounting for the reduced white blood cell count (Figure 4B–D). Similar amelioration of the CML blast crisis was also observed in the spleens of KO-CML mice, including reversed splenomegaly (Figure 4E) and decreased infiltration (Figure 4F and G). Correspondingly, loss of VDR significantly reduced BCR::ABL1-induced lethality, even in the secondary transplanted mice (Figure 4H; Supplementary Figure S4H).

Furthermore, VDR deficiency led to a lower frequency of leukemia stem cells (LSCs; GFP^+^Lin^−^Sca-1^+^c-Kit^+^) in BM (Figure 4I). This was further confirmed by the substantial reduction in the colony-forming cell (CFC)/replating capacity of LSCs from KO-CML mice (Figure 4J and K). Consistent with findings in CML cells *in vitro*, KO-CML mice exhibited prominent activation of DDR signaling and reduced DDIT4 expression, accompanied by a remarkable increase in cellular senescence in BM compared with WT-CML mice (Supplementary Figure S4I–K). These results demonstrated that VDR plays important roles in the pathogenesis of BCR::ABL1-driven CML.

### VDR deletion is an effective therapeutic strategy for overcoming TKI resistance in CML

**Figure 5:**
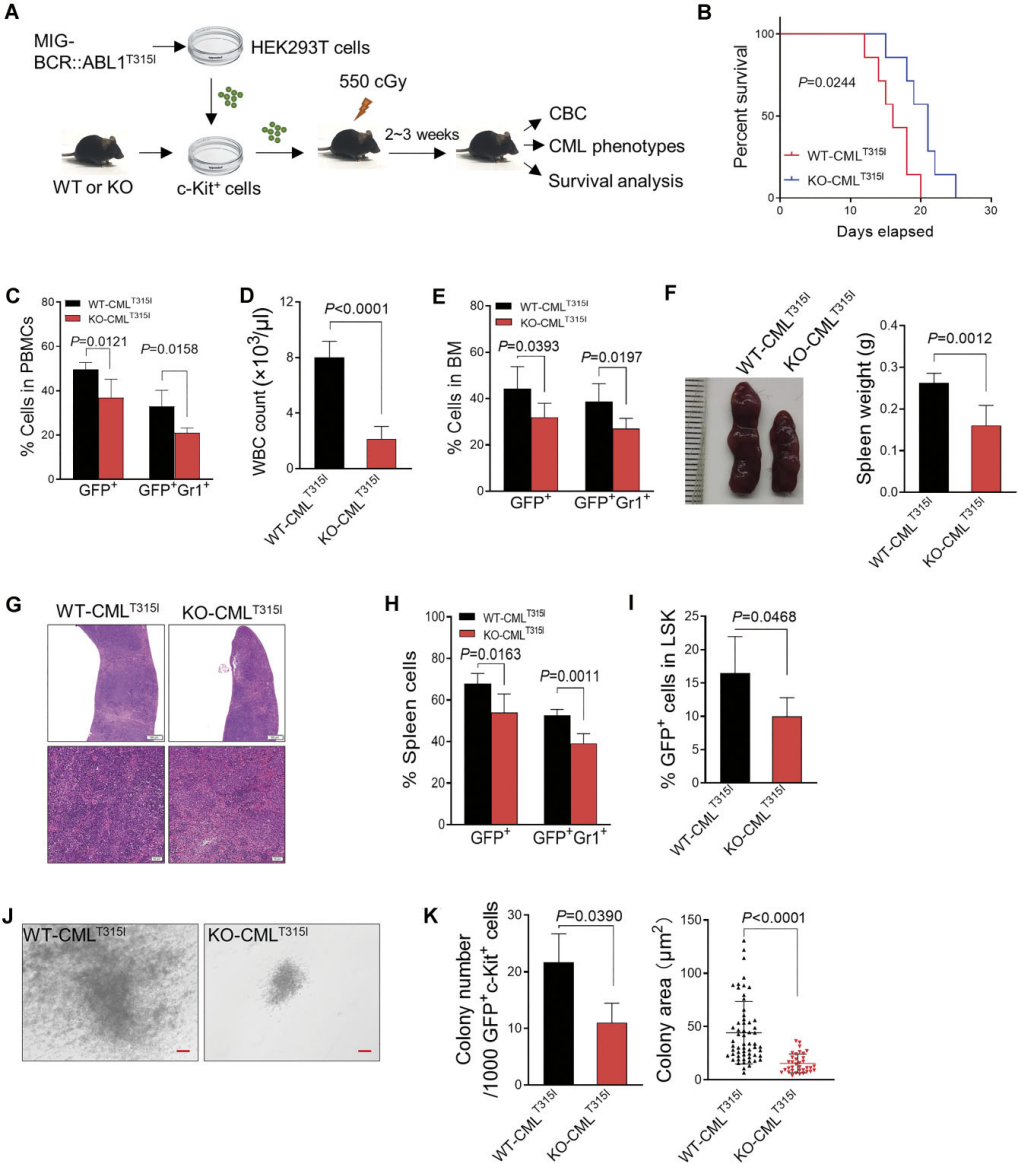
VDR deletion is an effective therapy for BCR::ABL1^T315I^-induced CML. (**A**) Experimental procedure for the assessment of the *in vivo* effect of VDR deficiency on BCR::ABL1^T315I^-driven CML (CML^T315I^) mice. CBC represents complete blood count. (**B**) Kaplan–Meier survival curves of the indicated CML mice as in **A** (*N* = 7). *P* values were determined by the log-rank (Mantel–Cox) test. (**C**) Quantification of the percentage of GFP^+^ cells and GFP^+^Gr1^+^ cells in PBMCs from the mice described in **A** (*N* = 5). (**D**) WBCs in peripheral blood were measured 14 days after transplantation from mice as in **C** (*N* = 5). (**E**) Quantification of the percentage of GFP^+^ cells and GFP^+^Gr1^+^ cells in the BM from the indicated mice measured by flow cytometry (*N* = 5). (**F**) Representative spleen of the indicated CML^T315I^ mice described in **A**. Statistical analysis of spleen weight is shown on the right (*N* = 6). (**G**) Representative hematoxylin and eosin (H&E) staining of spleens from the indicated CML^T315I^ mice 14 days after transplantation. Scale bar, 500 μm (upper) or 50 μm (lower). (**H**) Quantification of the percentage of GFP^+^ cells and GFP^+^Gr1^+^ cells in the spleen from the indicated mice measured by flow cytometry (*N* = 5). (**I**) Quantification of the percentage of GFP^+^LSK (Lin^–^Sca1^+^c-Kit^+^) in the BM from the indicated mice measured by flow cytometry (*N* = 5). (**J**) Representative colonies of GFP^+^c-Kit^+^ BM cells from the indicated CML^T315I^ mice. Scale bar, 10 μm. (**K**) Quantification of the colony number and colony area in the indicated group as in **J**. All *P* values were determined by an unpaired two-tailed Student's *t*-test except where indicated. See also Supplementary Figure S5.

Considering that the upregulated VDR expression and the inhibition of cell proliferation upon VDR knockdown in CML were independent of BCR::ABL1 mutations, we investigated whether inhibiting VDR could overcome the resistance of CML to TKIs *in vivo*. We established a similar CML mouse model with an IM-resistant BCR::ABL1^T315I^ mutation (referred to as CML^T315I^ mice) (Figure 5A) and found that VDR deletion significantly enhanced the survival of CML^T315I^ mice, which died 20 days after model establishment (Figure 5B), even in secondary transplanted mice (Supplementary Figure S5). KO-CML^T315I^ mice exhibited a remarkable reduction in white blood cell count and frequencies of leukemic cells (GFP^+^Gr1^+^) in peripheral blood and BM compared to WT-CML^T315I^ mice (Figure 5C–E). In addition, VDR deficiency also led to the substantial amelioration of the CML blast crisis in the spleen, as indicated by reversed splenomegaly and decreased infiltration (Figure 5F–H).

The frequencies of LSCs (GFP^+^Lin^−^Sca-1^+^c-Kit^+^) were significantly reduced upon VDR deletion (Figure 5I), although they showed similar engraftments (data not shown). In line with the *in vivo findings*, a reduced number and size of colonies were also observed in LSCs from KO-CML^T315I^ mice (Figure 5J and K), suggesting that loss of VDR efficiently repressed the proliferation of LSCs.

## Discussion

In this study, we identified VDR as a potent target for CML. VDR knockdown suppresses the proliferation of CML cells independent of BCR::ABL mutations and reduces LSCs. Although VDR is upregulated by the CML-driven gene BCR::ABL1 via transcriptional activation or protein stability that is dependent on the corresponding mutations, inhibiting VDR is sufficient to inhibit CML cell proliferation driven by BCR::ABL1 independent of its mutations *in vitro* and *in vivo*.

Although BCR::ABL1 inhibition led to a decrease in *VDR* mRNA expression, *VDR* mRNA levels were unchanged or mildly increased in the CML mouse model and Ba/F3 cells with ectopic expression of BCR::ABL1 and CML patients. Furthermore, BCR::ABL1 enhanced the protein stability of VDR. These findings indicated that BCR::ABL1 regulates *VDR* mRNA expression and stabilizes VDR. It is possible that the accumulated VDR provides negative feedback to suppress the transcriptional activity of VDR, leading to its low basis of mRNA expression in CML cells. It has been reported that VDR undergoes multiple posttranslational modifications (phosphorylation, SUMOylation, and ubiquitination) that mediate the degradation of VDR (Zhi et al., 2011; Wei et al., 2018). Although BCR::ABL1 is a fusion protein with kinase activity, we did not observe the interaction between BCR::ABL1 and VDR via co-IP assays (data not shown). Therefore, BCR::ABL1 may interact with and activate an unknown protein that mediates posttranslational modification of VDR to protect it from degradation.

Our data showed that VDR was also highly expressed in BCR::ABL1^T315I^-driven CML mice and Ba/F3 cells. However, BCR::ABL1^T315I^ upregulated its mRNA expression but not its protein stability, suggesting that BCR::ABL1 and its mutations regulate VDR expression via different regulatory mechanisms. This was further confirmed by the distinct VDR expression in other BCR::ABL1 mutation-driven Ba/F3 cells.

DNA damage and compromised DNA repair lead to suppression of tumor development through the induction of apoptosis, possibly with contributions by cell cycle arrest and cell senescence (Jin and Robertson, 2013). Consistent with one previous study showing that vitamin D/VDR axis regulates DNA repair during oncogene-induced senescence (Graziano et al., 2016), we found that VDR knockdown led to DDR and subsequent senescence, accounting for the proliferative inhibition in CML cells. Indeed, VDR regulates the expression of the DNA repair genes *RAD50* and *ATM* in response to DNA damage (Ting et al., 2012).

DDIT4, also known as regulated in development and DNA damage response-1 (Redd1), is highly expressed and associated with a worse prognosis in acute myeloid leukemia and solid tumors (Pinto et al., 2017). Our data demonstrated that *DDIT4* is the target gene of VDR in CML cells, which is responsible for the DNA damage and senescence mediated by VDR. In line with our findings, DDIT4 has recently been reported to be downregulated during oxidative stress- and UV-induced senescence, and its overexpression reverses this cellular senescence (Lee et al., 2022). Therefore, our data suggest that the VDR/DDIT4 axis plays important roles in CML cell proliferation via DNA damage repair, and its deficiency leads to DNA damage accumulation and senescence.

Alternatively, it is also possible that DDIT4 directly regulates p53 signaling to induce senescence. p53 is a central regulator of the DDR and genomic integrity, and its activation induces the transcription of its critical effector p21 to drive fundamental cellular processes, including senescence (Vousden and Prives, 2009; Brady et al., 2011). DDIT4 is a crucial inhibitor of mTOR that exerts different effects on cell proliferation and senescence (Brugarolas et al., 2004; DeYoung et al., 2008), and its knockdown exerts mTORC1-dependent control to activate p53 signaling (Vadysirisack et al., 2011; Du et al., 2018). Consistently, we observed that VDR knockdown led to mTOR signaling activation in K562 cells (data not shown) in parallel with p53 signaling activation. This may be due to reduced DDIT4 expression.

Notably, our data also showed that targeting VDR reduced CML-LSCs. Residual leukemic stem cells are the major barrier against CML therapy, leading to disease relapse and progression (Braun et al., 2020). Indeed, VDR has been reported to regulate HSPC expansion and survival via IL-8 activity (Cortes et al., 2016). On the other hand, VDR functions as a critical modulator of HSC trafficking via the neuronal control of the HSC niche (Kawamori et al., 2010). In line with these findings, we also elucidated a role for VDR in LSC expansion, and the deficiency of VDR led to reduced LSCs in CML. Given the similar features of the quiescence of stem cells and cellular senescence characterized by limited proliferative activity (Hernandez-Segura et al., 2018), VDR knockdown may also trigger senescence in LSCs, which contributes to the impaired self-renewal of LSCs.

It is evident that senescence-inducing therapies are a promising strategy for cancer (Wang et al., 2020). Senescence not only directly controls tumor growth via proliferative inhibition but also inhibits tumor progression via activation of immunity mediated by senescence-associated secretory phenotype factors produced by senescent cells. On the other hand, senescence-inducing therapies can also be combined with senolytic treatments (senolytic agents) as a ‘one-two punch’ approach to cancer therapy (Wang et al., 2022). Recently, this ‘one-two punch’ strategy by sequential use of one-two punch prosenescence and senolytic therapy has been successfully used to kill cancer cells (Wang et al., 2019). Our data provide evidence that inhibiting VDR triggers senescence in CML cells, which leads to proliferative inhibition *in vitro*. Loss of VDR exhibited mild but significant amelioration of the disease burden and progression in primary CML mice. This may be due to the accumulation of senescent leukemia cells *in vivo*. Therefore, our study identified VDR as a potential target for prosenescence therapy, the combination of which with senolytic therapy can provide remarkable benefit for CML therapy.

In summary, we demonstrated that VDR is needed for BCR::ABL1-driven CML progression and that targeting VDR has the potential to suppress the proliferation of CML cells independent of BCR::ABL mutations and eliminate LSCs.

## Materials and methods

### CML mouse model

C-Kit^+^ BM cells from WT and KO mice were transduced twice with the corresponding retrovirus in the presence of interleukin-3 (IL-3), interleukin-6 (IL-6), and stem cell factor (SCF). WT recipient mice were subjected to 5.5 Gy X irradiation and then injected with these infected c-Kit^+^ BM cells (2 × 10^6^ per mouse) via tail vein injection. After transplantation, the incidence of CML in the recipient mice was observed and recorded. Peripheral blood was collected from the tail vein into ethylenediaminetetraacetic acid (EDTA)-treated tubes and analyzed using a BC-5150 (Mindray). For secondary transplantation, all BM cells and splenic cells from the same group were combined, and the percentage of GFP^+^ cells was determined by FACS analysis. An equal number of total GFP^+^ cells were transplanted into irradiated secondary recipients. Mice were monitored for survival. All animal studies were performed in accordance with the Guidelines for the Care and Use of Laboratory Animals and were approved by the Institutional Animal Care and Use Committees at Shandong University.

### SA-β-gal staining

β-galactosidase (β-gal) was assayed using a kit from Beyotime Biotechnology. Cells were fixed for 15 min in β-gal fixative, washed with phosphate buffer saline (PBS), and stained in β-gal staining solution at 37°C until β-gal staining became visible in either experimental or control plates. Cells were washed in PBS, and the numbers of β-gal-positive cells (blue staining) were counted in random fields in each of the triplicate wells.

### Data sharing statement

Microarray data are available at GEO under accession number GSE233861.

### Statistical analysis

Statistical analyses were performed with an unpaired two-tailed Student's *t*-test, except where indicated otherwise, using Prism (GraphPad). For the cell proliferation rate, the *P* value was determined by a two-way ANOVA. For the survival curves of mice, *P* values were determined by the log-rank (Mantel–Cox) test. Data are presented as mean ± SD from three independent experiments except where indicated otherwise. The *P* value was determined by an unpaired two-tailed Student's *t*-test. *P < 0.05; **P < 0.01; ***P < 0.001; ****P < 0.0001. *P* values < 0.05 were considered significant.

Additional methods and detailed information on the antibodies, reagents, and primer sequences are provided in the supplementary information.

## Supplementary Material

mjad066_Supplemental_Files

## Notes

**Author contributions**: B.Z. designed and guided the research; Y.X., W.Q., and C.Z. performed the experiments; Y.X., Z.L., and B.Z. analyzed the data; Y.X. and B.Z. wrote the original draft; Y.L., J.G., C.L., and B.Z. reviewed and edited the manuscript. All authors have read and agreed to the published version of the manuscript.
