# Supplementary material for: Loss of the vitamin D receptor triggers senescence in chronic myeloid leukemia via DDIT4-mediated DNA damage
Source: J Mol Cell Biol. 2023 Oct 25;15(10):mjad066. doi: 10.1093/jmcb/mjad066 (PMC11190374; doi:10.1093/jmcb/mjad066)
Supplement: mjad066_Supplemental_Files [file mjad066_supplemental_files.zip › Supplementary material.docx]

**Loss of Vitamin D receptor triggers senescence in chronic myeloid leukemia via DDIT4-mediated DNA damage**

Yan Xu^1,2,3^, Wentao Qi^1,2,3^, Chengzu Zheng^1,2,3^, Yuan Li^1,2,3^, Zhiyuan Lu^4^, Jianmin Guan^5^, Chunhua Lu^1,2^, Baobing Zhao^1,2,3#^

^1^Key Lab of Chemical Biology (MOE), School of Pharmaceutical Sciences, Cheeloo College of Medicine, Shandong University, Jinan, Shandong, 250012, China

^2^NMPA Key Laboratory for Technology Research and Evaluation of Drug Products, School of Pharmaceutical Sciences, Cheeloo College of Medicine, Shandong University, Jinan, Shandong, 250012, China

^3^Department of Pharmacology, School of Pharmaceutical Sciences, Cheeloo College of Medicine, Shandong University, Jinan, Shandong, 250012, China

^4^ School of Pharmaceutical Sciences & Institute of Materia Medica, Shandong First Medical University & Shandong Academy of Medical Sciences, Jinan, Shandong, 250012, China

^5^Department of Hematology, Heze Municipal Hospital, Heze, Shandong, 274031, China

^#^Correspondence to:

Baobing Zhao, Ph.D., Department of Pharmacology, School of Pharmaceutical Sciences, Shandong University, 44 W Wenhua Road, Jinan, Shandong, P.R.China, 250012; [baobingzh@sdu.edu.cn](mailto:baobingzh@sdu.edu.cn); TEL/FAX: +86-531-88382176.

**Supplementary Files:**

1. Supplementary Methods

2. Supplementary Tables S1-3 with legends

3. Supplementary Figures S1-5 with legends

**Supplementary Methods**

**Reagents**

Imatinib and Ponatinib were purchased from Selleck (Shanghai, China) and dissolved in DMSO at different concentrations. Cycloheximide (CHX) were purchased from MCE and dissolved in water. The stock solutions were stored at -80°C.

**Patient samples**

Bone marrow (BM) samples from CML patients and healthy donors without any malignant bone marrow disorder were obtained at the Heze Municipal Hospital in China. Informed consent was obtained in accordance with the Declaration of Helsinki. Acquisition of bone marrow samples was performed with the informed consent of the patients. The use of human tissues was approved by the Medical Institutional Ethics Committee of Heze Municipal Hospital, China. Bone marrow mononuclear cells were isolated by density gradient centrifugation using lymphocyte separation medium (Haoyang, China).

**Cell culture**

The Ba/F3 cells obtained from Chinese Academy of Medical Sciences was maintained in RPMI-1640 (Basal Media) supplemented with 10% fetal bovine serum (FBS) (Biological Industries) containing IL-3; their counterparts transformed with BCR::ABL1 or various BCR::ABL1 mutations were maintained in RPMI-1640 supplemented with 10% FBS. K562 cells was purchased from Chinese Academy of Medical Sciences, and cultured in RPMI 1640 medium supplemented with 10% FBS. KBM5 and KBM5-T315I cells were kindly provided by Jingxun Pan (Sun Yat-sen university), and cultured in IMDM (Thermo Fisher Scientific) supplemented with 10% FBS. HEK293T cells were obtained from Chinese Academy of Medical Sciences and cultured in DMEM (Basal Media) with 10% FBS. All cell lines were cultured at 37 °C in a humidified atmosphere of 5 % CO_2_.

**Mice**

VDR knockout mice (C57BL/6J background) were obtained from the Jackson Laboratory. All subsequent mice used in this study were generated from mating with littermates. The VDR genotype was determined by PCR analysis. All mice were bred in an equipped animal facility with the temperature at 20-25°C, a 12 hours light-dark cycle and ad libitum access to regular chow diet and water. Experiments were performed on age- and sex-matched cohorts. All animal studies were performed in accordance with the Guidelines for the Care and Use of Laboratory Animals and were approved by the Institutional Animal Care and Use Committees at Shandong University.

**Plasmids construction and cell transduction**

ShRNA oligonucleotides and overexpressed plasmid were designed and synthesized (Supplementary Table S2). The retrovirus supernatant was packaged in HEK293T cells by co-transfecting with object plasmid and packaging plasmid. Then viral supernatant was collected after transfection for 48 and 72 hours and purified with 0.45-μm filter. The cells were transfected with viral supernatant and polybrene (8 μg/mL) by centrifugation (1800 rpm, 90 min, 37°C).

**Colony-forming assay**

GFP^+^c-Kit^+^ cells from BM of WT-CML or KO-CML mice were obtained using c-Kit^+^ selection cocktail and cell sorting. 1000 c-Kit^+^ cells were mixed with the stem cell medium (H3534, STEMCELL), and then continuously incubated at 37°C in 5% humidified CO_2_. After incubation for 14 days, the colonies were counted.

**Flow cytometric analysis**

Cell cycle analysis. Cells were collected and rinsed with cold PBS and fixed in 75% ethanol at -20°C for at least 6 hours. Subsequently, the cells were rinsed again with cold PBS for two times, incubated with RNase (10 μg/mL) at 37°C for 30 min and stained with propidium iodide (PI; 50 μg/mL). Flow cytometry was used to examine the percentages of cells at different cell cycle stages.

Murine hematopoietic cells assay. Single-cell suspensions of blood, BM and spleen were prepared and stained with a ‘‘cocktail’’ of antibody conjugates for 20 min at room temperature in flow cytometry buffer. Data were collected on a FACS and were analyzed with NovoExpress software. Information of antibodies for flow cytometry assay are detailed in (Supplementary Table S3).

**Luciferase reporter assays**

HEK293T cells were transiently co-transfected with a luciferase reporter construct bearing the -2000 *DDIT4* promoter and VDR plasmid or empty vector for 24 hours. After 24 hours, cells were lysed with reporter lysis buffer and subjected to luciferase reporter assays according to manufacturer’s instructions (Promega).

**mRNA extraction and Quantitative RT-PCR**

Total RNA was extracted using the Trizol reagent according to the manufacturer’s instructions (Invitrogen). RNA quality and quantity were determined using a Nano Drop and Agilent 2100 bioanalyzer (Thermo Fisher Scientific). RNA was reverse transcribed into cDNA using RT reagent kit (TaKaRa, Japan). A SYBR Green PCR kit was used for quantitative real-time PCR and results were quantified with an Applied Biosystem System (ABI) with appropriate primers. The human housekeeping gene 18S was used as the RNA-loading control. Gene expression was determined by the delta CT method (2^-(∆∆Ct)^). ∆Ct = (Ct target gene - Ct housekeeping). The primer sequences were listed in (Supplementary Table S2).

**Immunoblotting**

Cells were lysed in RIPA buffer (Beyotime) with protease and phosphatase inhibitor mixture (Beyotime) for 30 min, then centrifuged at 12000 rpm, 4 °C. The protein concentration was quantified by bicinchoninic acid (BCA) protein kit (Beyotime). The protein expression levels were determined by staining with primary antibodies (Supplementary Table S3).

**EdU cell proliferation assay**

EdU analysis of K562 cells were performed using the Cell-Light EDU Apollo567 In vitro Kit (RIBBIO). 1 ×10^5^ cells were harvested and labeled with 50 µM EdU in medium for 2 hours in 37°C incubator. Cells were washed once with 1×PBS and the pellets were resuspended in 100 μL 4% PFA for 30 minutes at room temperature. After fixation, cells were washed with 2 mg/mL glycine for 5 minutes and then permeabilization with PBS containing 0.5% Triton X-100. After the 10-minute incubation period, 100 μL Apollo Stain mix was added to each sample and incubated for 30 minutes at room temperature. Wash three times with PBS containing 0.5% Triton X-100, DNA staining, cover slip, and observed by fluorescence microscope.

**Chromatin immunoprecipitation**

ChIP assays were performed using a SimpleChIP Enzymatic Chromatin IP Kit (Cell Signaling Technology) according to the manufacturer’s protocol.

**Supplementary Tables**

**Supplementary Table S1. GO enrichment analysis of the differentially expressed genes from RNA sequencing of K562 cells transduced with retrovirus encoding VDR shRNA.**

**Supplementary Table S2. Primer sequences used in this study.**

| **Gene** | **Oligonucleotides** |
| --- | --- |
| Human-VDR-shRNA-2 | Forward: 5’-CCGGCCTCCAGTTCGTGTGAATGATCTCGAGATCATTCAC  ACGAACTGGAGGTTTTTG-3′  Reverse: 5’-AATTCAAAAACCTCCAGTTCGTGTGAATGATCTCGAGATCA  TTCACACGAACTGGAGG-3′ |
| Human-VDR-shRNA-4 | Forward: 5’-CCGGATGAAGCGGAAGGCACTATTCCTCGAGGAATAGTGC  CTTCCGCTTCATTTTTTG-3′  Reverse: 5’-AATTCAAAAAATGAAGCGGAAGGCACTATTCCTCGAGGAAT  AGTGCCTTCCGCTTCA-3′ |
| Mouse-VDR-shRNA-2 | 5’-aaaaCCTGAGATCAATCACATTTAAgtcgacCTCGAGTTAAATGT  GATTGATCTCAGG-3′ |
| Mouse-VDR-shRNA-3 | 5’-aaaaCGTGGACATTGGCATGATGAAgtcgacCTCGAGTTCATCATG  CCAATGTCCACG-3′ |
| Human-HA-VDR | Forward: 5’- GAGGAGAATCCTGGCCCAccgTTCGAAATGTACCCAT  ACGACGTCCCAGACTACGCTatggaggcaatggcggccagc-3′  Reverse: 5’- tattaggtccctcgacgaattctcaggagatctcattgcc  aaacac -3′ |
| Human-Flag-DDIT4 | Forward: 5’-GTCGAGGAGAATCCTGGCCCAccgTTCGAAATGGATTACA  AGGATGACGACGATAAGatgcctagcctttgggaccgc-3’  Reverse: 5’- tacgaagttattaggtccctcgacgaattctcaacactcc  tcaatgagcagctg-3’ |
| Human-DDIT4-promotor | Forward: 5’-agaacatttctctatcgataggtaccAGTTGGTTGAGTC  TCTCATCAGTAC-3′  Reverse：5’- ttgatgagtcagtgcccgggctagcTTACCAAAGTTGAT  TTTATGTGTAG -3′ |
| Human-VDR primer | Forward: 5’- GACCTCACAGAAGAGCACCC -3′  Reverse: 5’- CGTTCCGGTCAAAGTCTCCA-3′ |
| Human-DDIT4 primer | Forward: 5’-CGCCTGGACTCACGACTCTG -3′  Reverse: 5’-AAGCCAGTGCTCAGCGTCAG -3′ |
| Human-BCR::ABL1 primer | Forward: 5’- TCCACTCAGCCACTGGATTTAA -3’  Reverse: 5’- TGAGGCTCAAAGTCAGATGCTACT -3’ |
| Mouse-18S primer | Forward: 5’-GCAATTATTCCCCATGAACG-3’  Reverse: 5’-GGCCTCACTAAACCATCCAA-3’ |

**Supplementary Table S3. Antibodies and commercial reagents used in this study.**

| **Antibodies/Regents** | **Source** | **Catalog Number** |
| --- | --- | --- |
| VDR antibody | CST | 12550S |
| BCR::ABL1 antibody | CST | 2862S |
| HSC70 antibody | Proteintech | 10995-1-AP |
| P-BCR::ABL1 antibody | CST | 2861S |
| HA-tag antibody | Affinity | T0050 |
| GAPDH antibody | Abcam | ab9385 |
| P-p53 (S15) antibody | CST | 9284S |
| P21 antibody | Proteintech | 10355-1-Ap |
| phospho-Histone H2A.X (Ser139) antibody | CST | 9718T |
| DDIT4 antibody | Affinity | DF8353 |
| APC- anti-mouse- TER119 | eBioscience | 47-5921-80 |
| APC- anti-mouse- CD3ε | Biolegend | 100312 |
| PE- anti-mouse- CD11b | Biolegend | 101207 |
| FITC- anti-mouse- B220 | Biolegend | 103206 |
| PB- anti-mouse- Ly-6G/Ly-6C | Biolegend | 108430 |
| PE- anti-mouse- CD41 | Biolegend | 133906 |
| PB- anti-mouse- Lineage | Biolegend | 133306 |
| FITC- anti-mouse- Sca-1 | eBioscience | 11-5981-85 |
| APC- anti-mouse- CD117 | eBioscience | 17-1171-83 |
| Hexadimethrine bromide | Sigma | H9268 |
| Dual-Luciferase® Reporter Assay System | Promega | REF.E1910 |
| PrimerScript RT reagent | Takara | RR047A |
| SYBR Premix Ex Taq | Takara | RR420A |
| Cell-Light EDU Apollo567 In vitro Kit | RIBBIO | C10310-1 |

**Supplementary Figure Legends**


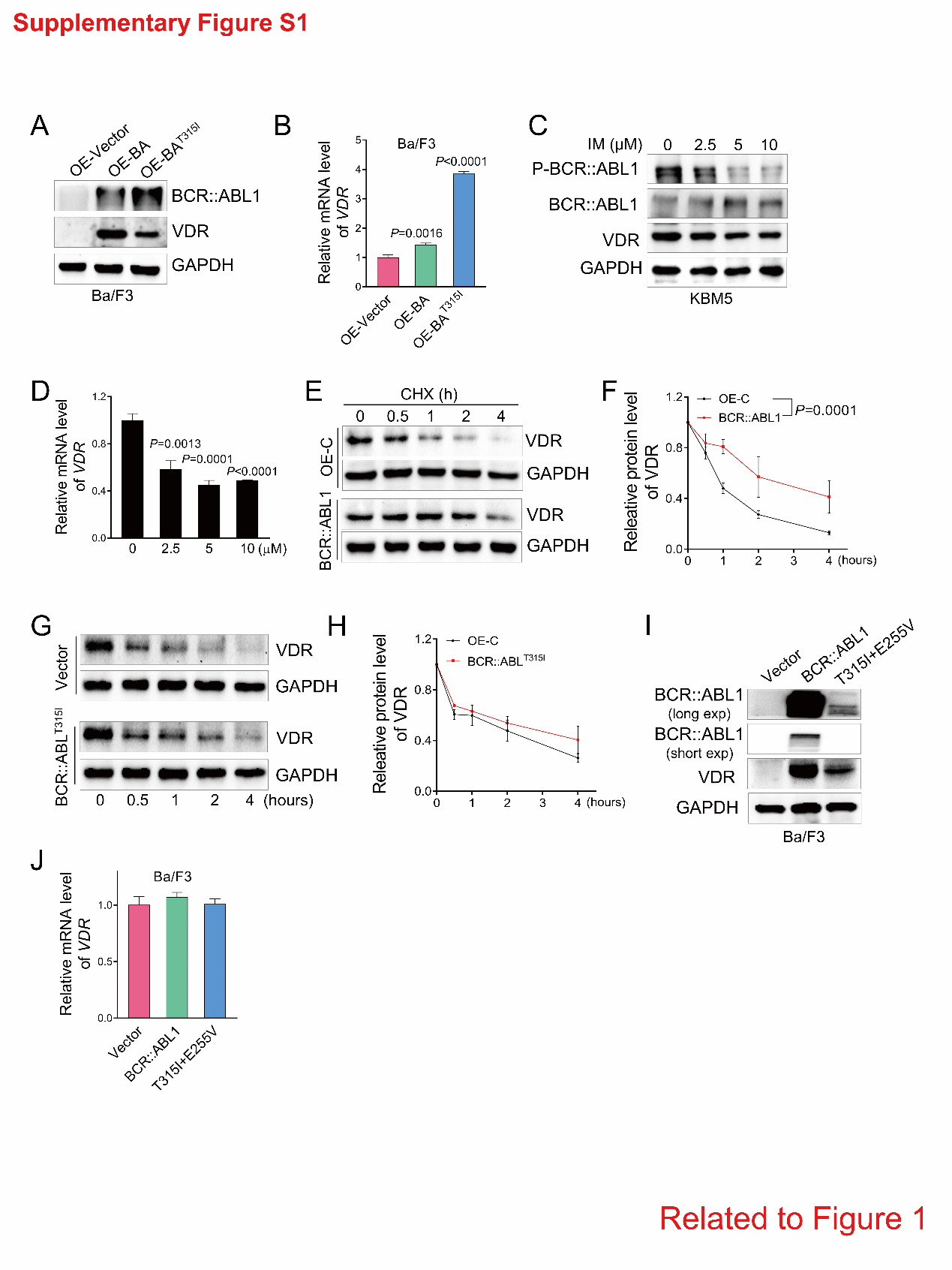


**Supplementary Figure S1. BCR::ABL1 regulated VDR expression in CML. (A)** Immunoblotting analysis of the VDR and BCR::ABL1 protein levels in the Ba/F3 cells transduced with retroviral constructs encoding BCR::ABL1^P210/T315I^ (OE-BA, OE-BA^T315I^) or empty vector (OE-Vector). GAPDH was used as a loading control. **(B)** Quantification of mRNA expression of *VDR* in the Ba/F3 cells as in A. **(C)** Immunoblotting analysis of indicated protein levels in KBM5 cells with the treatment of imatinib (IM) for 10 hours. GAPDH was used as a loading control. **(D)** Quantification of mRNA expression of *VDR* in cells as in C. (**E-F**) Immunoblotting analysis of VDR protein in Ba/F3 cells transduced with retroviruses encoding BCR::ABL1 or empty vector (OE-C) upon cycloheximide (CHX, 10 μM) treatment. VDR level was normalized to the change of GAPDH. Data were presented as mean ± SD from three independent experiments. *P* value was determined by two-way ANOVA. (**G-H**) Immunoblotting analysis of VDR protein in Ba/F3 cells transduced with retroviruses encoding BCR::ABL1^T315I^ or empty vector (OE-C) upon cycloheximide (CHX, 10 μM) treatment. VDR level was normalized to the change of GAPDH. Data were presented as mean ± SD from three independent experiments. *P* value was determined by two-way ANOVA. (**I**) Immunoblotting analysis of the VDR and BCR::ABL1 protein levels in Ba/F3 cells transduced with retroviral constructs encoding BCR::ABL1, BCR::ABL1^T315I+E255V^ or empty vector. GAPDH was used as a loading control. **(J)** Quantification of mRNA expression of *VDR* in cells as in I. All *P* values were determined by unpaired two-tailed Student’s t-test except where indicated otherwise. Data are presented as mean ± SD from three independent experiments. Related to Figure 1.


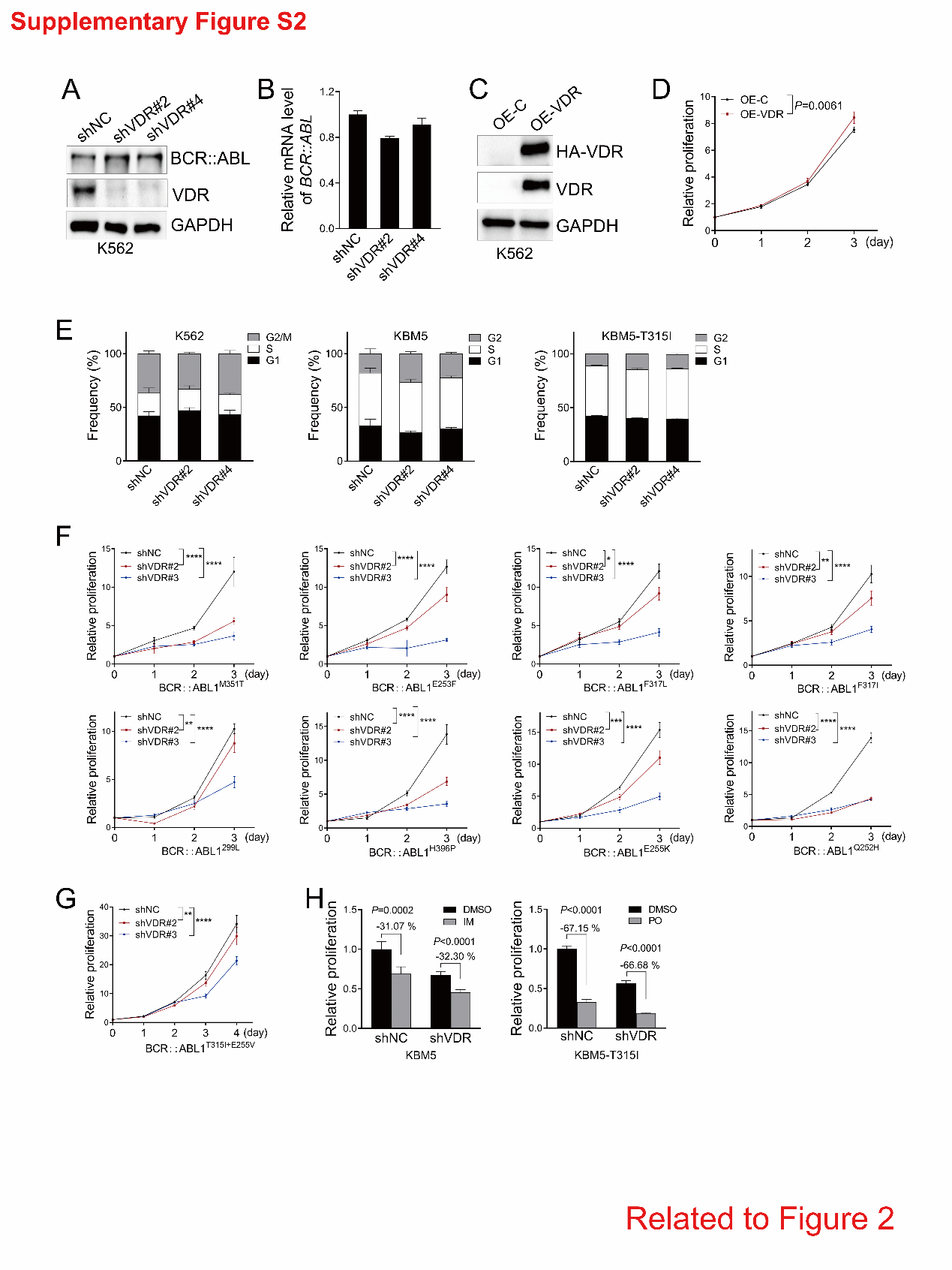
**Supplementary Figure S2. VDR regulated the proliferation of CML cells but without effects on cell cycle.** **(A)** Immunoblotting analysis of VDR and BCR::ABL1 protein levels in K562 cells transduced with retroviruses encoding indicated shRNA. shNC represents a non-targeting shRNA. GAPDH was used as a loading control. **(B)** Quantification of mRNA expression of *BCR::ABL* in cells as in A. **(C)** Immunoblotting analysis of VDR and HA-tag labeled VDR protein levels in K562 cells transduced with VDR plasmid or empty vector. GAPDH was used as a loading control. **(D)** Statistical analysis of cell proliferation in K562 cells as in C. Data were obtained from three independent experiments. *P* value was determined by two-way ANOVA. **(E)** Cell cycle was measured in the K562, KBM5 and KBM5-T315I cells transduced with retroviruses encoding indicated shRNA on day 2 (N=3). shNC represents a non-targeting shRNA. **(F)** Statistical analysis of cell proliferation in the BCR::ABL1 mutations Ba/F3 cells transduced with retroviruses encoding indicated shRNA. shNC represents a non-targeting shRNA. Data were obtained from three independent experiments. *P* value was determined by two-way ANOVA. * *P* < 0.05; ** *P* < 0.01; *** *P* < 0.001; **** *P*<0.0001. **(G)** Statistical analysis of cell proliferation in the BCR::ABL1^T315I+E225V^ mutation Ba/F3 cells transduced with retroviruses encoding indicated shRNA. shNC represents a non-targeting shRNA. Data were obtained from three independent experiments. *P* value was determined by two-way ANOVA. ** *P* < 0.01; *** *P* < 0.001. **(H)** Statistical analysis of cell proliferation in the KBM5 or KBM5-T315I cells transduced with retroviruses encoding indicated shVDR, and treated with Imatinib (IM) or Ponatinib (PO). shNC represents a non-targeting shRNA. Data were obtained from three independent experiments. All *P* values were determined by unpaired two-tailed Student’s t-test except where indicated otherwise. Data are presented as mean ± SD from three independent experiments. Related to Figure 2.


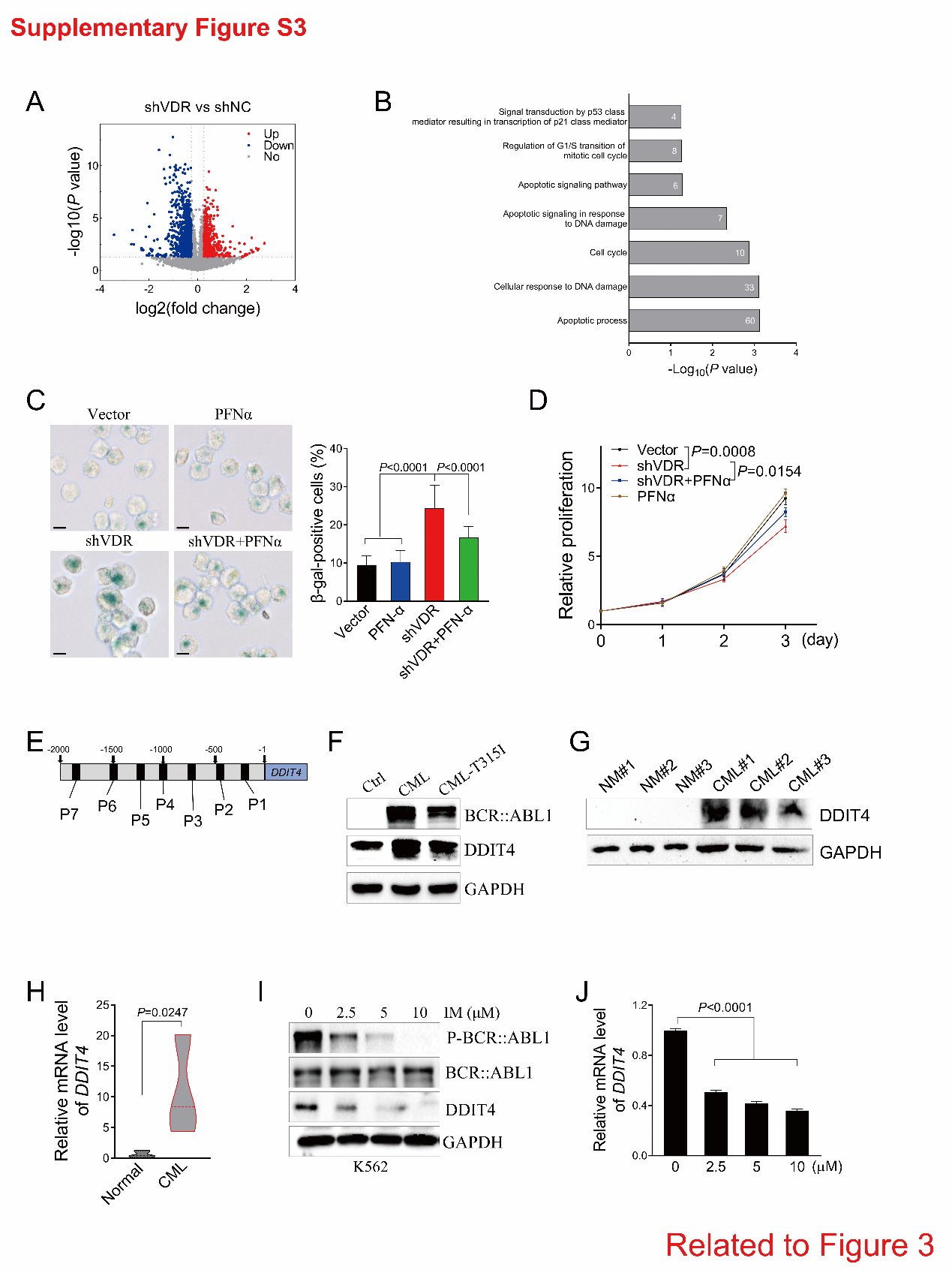
**Supplementary Figure S3. Inhibition of p53 signaling rescued the phenotypes in VDR knockdown CML cells and BCR::ABL1 regulated DDIT4 expression. (A)** RNA-sequence analysis of K562 cells transduced with retroviruses encoding shVDR or shNC after 10 hours (N=3). shNC represents a non-targeting shRNA. The volcano plot of the differentially expressed genes (DEGs) were shown. Red dots represent upregulated genes, blue dots represent downregulated genes and grey dots represent unchanged genes. **(B)** DAVID pathway analysis of all the DEGs in K562 cells as in A. **(C)** P53 inhibitor partially rescued the VDR knockdown-induced senescence in K562 cells. Representative images of SA-β-gal staining were shown on the left. Quantification of the percentage of SA-β-gal positive cells in indicated group was shown on the right. Scale bar: 12.5 μm. **(D)** P53 inhibitor partially rescued the VDR knockdown-mediated proliferation inhibition in K562 cells. Statistical analysis of cell proliferation as in C. Data were obtained from three independent experiments. *P* value was determined by two-way ANOVA. **(E)** Representative *DDIT4* promoter region (up to 2000 bp from the start codon of *DDIT4*). Black blocks indicate fragments (P1 to P7) in the *DDIT4* promoter region amplified in ChIP–qPCR assays**.** P1: -167 ~ -345; P2: -394 ~ -500; P3: -780 ~ -932; P4: -933 ~ -1060; P5: -1155 ~ -1348; P6: -1403 ~ -1513; P7: -1770 ~ -1910. **(F)** Immunoblotting analysis of DDIT4 and BCR::ABL1 protein levels in the BM cells from indicated CML mice and corresponding control (Ctrl). GAPDH was used as a loading control. **(G)** Immunoblotting analysis of DDIT4 protein level in the bone marrow mononuclear cells from CML patients and healthy donors (normal, NM). GAPDH was used as the loading control that was the same as in Figure 1C. (**H**) Quantification of mRNA expression of *DDIT4* in the cells as in G. **(I)** Immunoblotting analysis of indicated protein levels in K562 cells with the treatment of imatinib (IM) for 10 hours. GAPDH was used as a loading control. **(J)** Quantification of mRNA expression of *DDIT4* in K562 cells treated with Imatinib as in I. All *P* values were determined by unpaired two-tailed Student’s t-test except where indicated otherwise. Data are presented as mean ± SD from three independent experiments. Related to Figure 3.


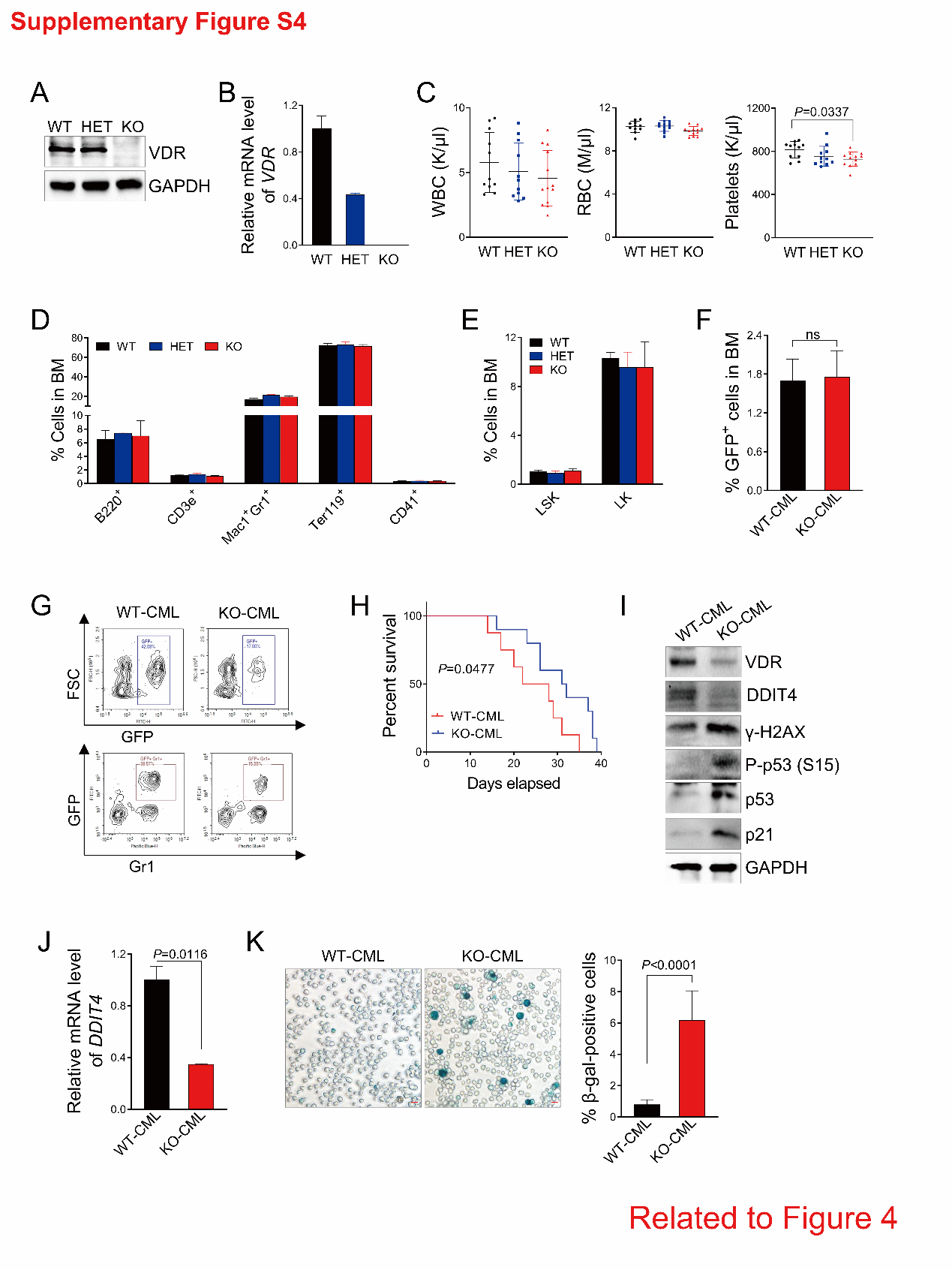
**Supplementary Figure S4. Effects of VDR deletion on normal hematopoiesis and CML disease. (A)** Immunoblotting analysis of VDR protein level from BM in the 8-week-old VDR^+/+^, VDR^+/-^ and VDR^−/−^ mice (namely WT, HET, and KO). GAPDH was used as a loading control. **(B)** Quantification of mRNA expression of *VDR* from BM as in A. **(C)** Peripheral blood chimerism analysis of WT, HET, and KO mice as in A. Each dot represents one mouse. White blood cell (WBC), Red blood cell (RBC). **(D)** Quantification of the frequency of B cells, T cells, Mac1^+^Gr1^+^ cells, Ter119^+^ and CD41^+^ cells in the BM from indicated group as in A (N=4). **(E)** Quantification of the frequency of LSK and LK cells in the BM from indicated group as in A (N=4). **(F)** Homing assay. C-Kit^+^ BM cells from WT or KO mice were transfected with retroviruses encoding BCR::ABL1 and then transplanted into CD45.1 recipient mice via tail vein injection. After 16 hours, BM cells from recipients were stained with CD45.2 antibody. Quantitative analysis of the percentage of CD45.2^+^ cells was shown (N=4). **(G)** Representative flow cytometry plots of GFP^+^ cells and GFP^+^Gr1^+^ cells in peripheral blood from WT-CML and KO-CML mice measured by flow cytometry. **(H)** BM and splenic cells isolated from WT-CML and KO-CML mice were transplanted into the secondary recipients via tail vein injection. Kaplan-Meier survival curves of secondary transplantation were shown (N=10). *P* values were determined by Log-rank (Mantel-Cox) test. **(I)** Immunoblotting analysis of indicated protein levels in the BM cells from indicated CML mice as in G. GAPDH was used as a loading control. **(J)** Quantification of mRNA expression of *DDIT4* in BM cells of the indicated CML mice as in G. **(K)** Representative SA-β-gal staining in cells as in G. Scale bars represent 20 μm. All *P* values were determined by unpaired two-tailed Student’s t-test except where indicated otherwise. Data are presented as mean ± SD from three independent experiments. Related to Figure 4.


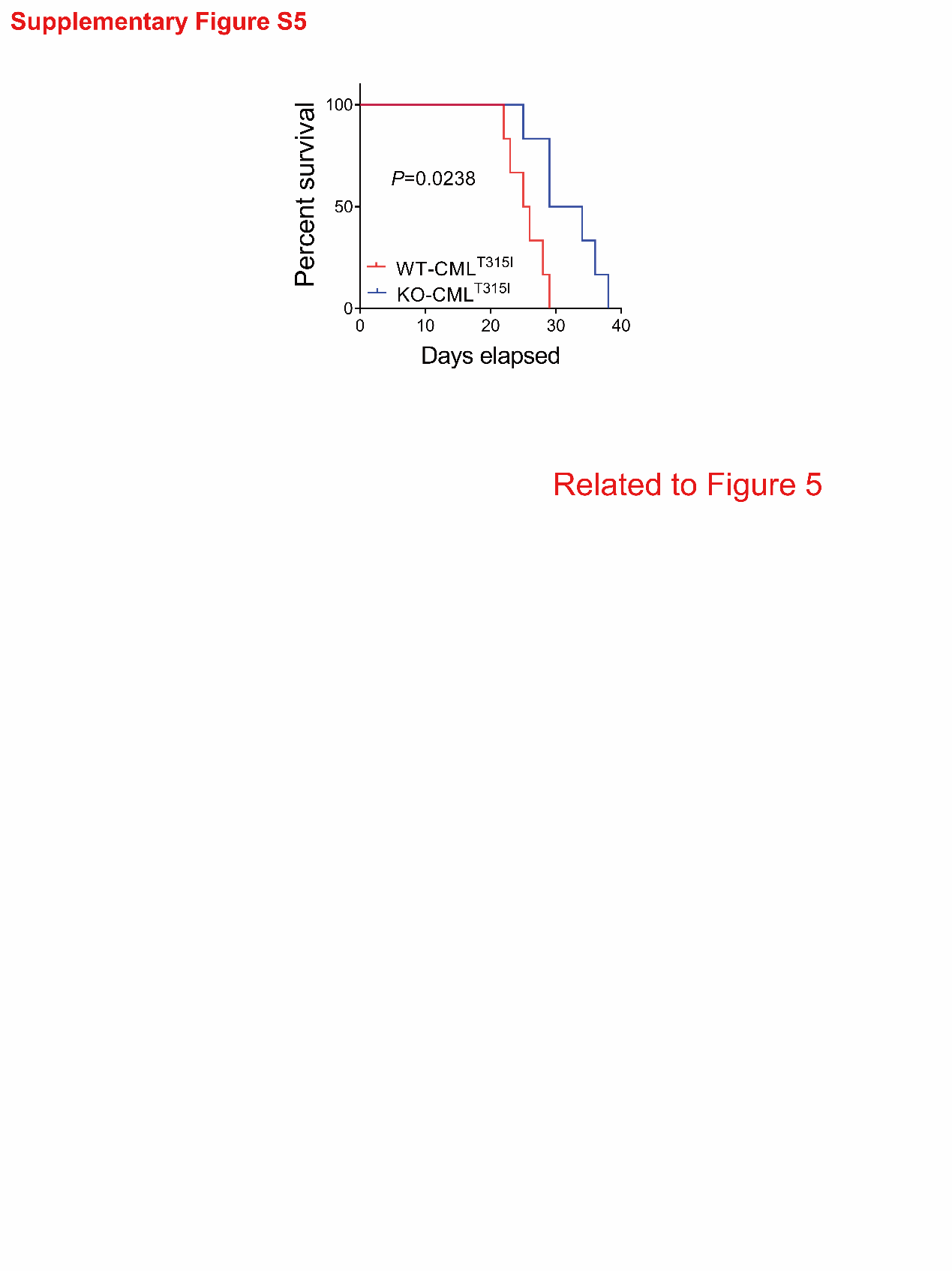
**Supplementary Figure S5. VDR knockout suppressed the survival of BCR::ABL1^T315I^-induced CML.** BM and splenic cells isolated from WT and KO CML^T315I^ mice were transplanted into the secondary recipients via tail vein injection. Kaplan-Meier survival curves of secondary transplantation were shown (N=6). *P* values were determined by Log-rank (Mantel-Cox) test. Related to Figure 5.
